# Development and validation of deep learning ECG-based prediction of myocardial infarction in emergency department patients

**DOI:** 10.1038/s41598-022-24254-x

**Published:** 2022-11-15

**Authors:** Stefan Gustafsson, Daniel Gedon, Erik Lampa, Antônio H. Ribeiro, Martin J. Holzmann, Thomas B. Schön, Johan Sundström

**Affiliations:** 1 Clinical Epidemiology Unit, Department of Medical Sciences, Uppsala University, Uppsala, Sweden; 2 Sence Research AB, Uppsala, Sweden; 3 Division of Systems and Control, Department of Information Technology, Uppsala University, Uppsala, Sweden; 4 Function of Emergency Medicine, Karolinska University Hospital, Stockholm, Sweden; 5 Department of Medicine, Solna, Karolinska Institutet, Stockholm, Sweden; 6 The George Institute for Global Health, University of New South Wales, Sydney, Australia

**Keywords:** Cardiology, Health care, Medical research, Diseases, Cardiovascular diseases, Machine learning

## Abstract

Myocardial infarction diagnosis is a common challenge in the emergency department. In managed settings, deep learning-based models and especially convolutional deep models have shown promise in electrocardiogram (ECG) classification, but there is a lack of high-performing models for the diagnosis of myocardial infarction in real-world scenarios. We aimed to train and validate a deep learning model using ECGs to predict myocardial infarction in real-world emergency department patients. We studied emergency department patients in the Stockholm region between 2007 and 2016 that had an ECG obtained because of their presenting complaint. We developed a deep neural network based on convolutional layers similar to a residual network. Inputs to the model were ECG tracing, age, and sex; and outputs were the probabilities of three mutually exclusive classes: non-ST-elevation myocardial infarction (NSTEMI), ST-elevation myocardial infarction (STEMI), and control status, as registered in the SWEDEHEART and other registries. We used an ensemble of five models. Among 492,226 ECGs in 214,250 patients, 5,416 were recorded with an NSTEMI, 1,818 a STEMI, and 485,207 without a myocardial infarction. In a random test set, our model could discriminate STEMIs/NSTEMIs from controls with a C-statistic of 0.991/0.832 and had a Brier score of 0.001/0.008. The model obtained a similar performance in a temporally separated test set of the study sample, and achieved a C-statistic of 0.985 and a Brier score of 0.002 in discriminating STEMIs from controls in an external test set. We developed and validated a deep learning model with excellent performance in discriminating between control, STEMI, and NSTEMI on the presenting ECG of a real-world sample of the important population of all-comers to the emergency department. Hence, deep learning models for ECG decision support could be valuable in the emergency department.

## Introduction

Emergency department care costs are high^1^ and rising^2^ in developed societies. Based on limited data in a chaotic environment, emergency medicine doctors must make quick decisions about patients’ probabilities for many diagnoses and risks. Diagnostic error is commonplace^3,4^, and there is a desperate need for emergency department decision support systems^5^.

The emergency department handling of myocardial infarctions is especially precarious. In the United States, myocardial infarctions are missed in the range of 10–50,000 per year at emergency departments^6^. At the other end, less than half of those hospitalized for a suspected myocardial infarction are eventually diagnosed with the condition^7^. The electrocardiogram (ECG) has long been used for diagnosing myocardial infarctions. Some large myocardial infarctions are accompanied by an elevation of the ST-segment of the ECG, giving them the name ST-elevation myocardial infarctions (STEMIs). Other myocardial infarctions, the non-ST-elevation myocardial infarctions (NSTEMIs), are often inconspicuous to the human eye on the ECG, and rely on other means of diagnosis.

Artificial intelligence (AI) with deep learning based models has shown much recent promise in ECG classification^8^, for common ECG diagnoses^9^ as well as for traits with unclear ECG diagnostic criteria or those not usually thought of as ECG diagnoses^10–12^. Even ECGs that appear normal to the human eye carry information useful for AI algorithms^12,13^. AI is very promising in the diagnosis of myocardial infarction^14,15^, but many studies have used limited^16,17^ or managed^14–18^ datasets. We postulate that AI ECG interpretation may be useful also in the most important population—all-comers to emergency departments.

Most of the studies above, as well as most studies on deep learning for arrhythmia classification^19^, detection of myocardial infarctions^20^ or other ECG-based tasks^21^ use models based on convolutional layers. We employed a ResNet^22^, which is a special convolutional neural network with skip connections. This structure allows use of deeper models with more layers, which was an essential breakthrough in deep learning. By deploying a standardized architecture, we aimed to ensure reproducibility and understanding of the model components. This is in contrast to prior work which use specialized, non-standard architectures^14,15^. Using a large real-world sample of patients presenting at emergency departments, we developed and validated a deep learning model for diagnosis of NSTEMI and STEMI on the presenting ECG.

## Results

Of the included total 492,226 ECGs from the emergency department visit, 5,416 (1.1%) were recorded with an NSTEMI, 1,818 (0.4%) with a STEMI and 484,992 (98.5%) without a myocardial infarction. Clinical characteristics of the study sample are presented in Table 1 and stratified for the data splits in Supplementary Table 2, and the patients’ age and admission date distributions are shown in Supplementary Figs. 1 and 2.

**Table 1:** Clinical characteristics of the study sample.

	Control	NSTEMI	STEMI
Number of ECGs	484,992	5416	1818
**Clinical characteristics at ED visit**			
Age	65.0 (47.0,78.0)	71.0 (62.0,81.0)	66.0 (57.0,77.0)
Male	47.3	65.4	73.7
Year	2013 (2010,2015)	2013 (2011,2015)	2013 (2011,2015)
**Presenting complaint**			
Chest pain	21.5	71.3	70.1
Difficulty breathing	14.5	12.3	5.9
Dizziness	7.1	0.7	1.2
Heart problems	2.0	1.3	1.9
Circulatory arrest	0.1	1.1	3.7
Cardiovascular diagnoses prior to ED visit*			
Myocardial infarction	8.4	26.1	16.2
Unstable angina	4.2	11.2	6.4
Ischemic heart disease	20.1	43.1	23.8
Stroke	9.1	11.2	7.4
Peripheral artery disease	7.0	12.4	7.2
Heart failure	15.8	20.6	9.6
Atrial fibrillation	19.9	15.8	8.7
Cardiovascular disease	59.0	70.8	52.2
**Drugs with > = 1 dispensation within one year prior to ED visit**			
Renin-angiotensin system inhibitors	33.0	51.8	36.9
Calcium channel blockers	17.9	29.3	21.1
Beta-receptor blockers	36.3	50.6	33.9
Mineralocorticoid receptor antagonists	6.3	6.2	3.0
Diuretics	27.6	34.2	19.4
Anti-arrhythmic drugs	1.7	0.4	0.6
Statins	23.7	41.9	24.3
Anticoagulants	12.8	8.8	5.6
Antiplatelets	27.8	48.8	28.9
Cardiac enzymes within ED visit or coronary care unit hospitalization**			
Troponin I measured	1.1	8.2	13.8
Max troponin I (ng/L)	29.7 (29.7,40.0)	2700.0 (610.0,10,150.0)	18,100.0 (3100.0,47,150.0)
Troponin T measured	38.2	87.2	87.6
Max troponin T (ng/L)	9.9 (5.0,19.0)	266.0 (93.0,814.0)	1900.0 (519.0,4680.0)
NTproBNP measured	8.7	21.5	17.1
Max NTproBNP (ng/L)	1340 (286,4300)	2940 (784,8720)	3120 (772,9220)
Main diagnoses at admission***			
Myocardial infarction	0.0	94.1	97.2
Unstable angina	0.2	4.1	1.6
Ischemic heart disease	1.3	94.6	97.2
Stroke	2.0	1.1	0.7
Peripheral artery disease	0.4	0.3	0.3
Heart failure	3.3	2.1	0.9
Atrial fibrillation	5.9	0.8	0.3
Cardiovascular disease	18.4	96.2	98.4
**Mortality after coronary care unit admission**			
30-day all-cause death	3.5	6.4	10.6
In-hospital all-cause death	2.6	5.5	9.2

Patient characteristics of coronary care unit admissions included in the study, by control/NSTEMI/STEMI outcome. Data are medians (quartiles) or percent.

*Prevalent disease based on any diagnosis position, inpatient and outpatient specialist care combined.

**Combining troponin and high-sensitive troponin laboratory measurements from regional laboratory databases and the SWEDEHEART database. Maximum of all available measurements within the time window reported. Done separately for troponin I and T.

***Primary diagnosis from inpatient specialist care and/or specialized outpatient care at time of the ED/CCU visit.

STEMI, ST-elevation myocardial infarction; NSTEMI, non-ST-elevation myocardial infarction; ED, emergency department; NTproBNP, N-terminal pro-B-type natriuretic peptide.

We conducted a preliminary analysis in a subset of the study sample including only patients that were admitted to the coronary care unit (CCU), i.e. omitting the large pool of controls with an emergency department visit without being transferred to the CCU. From this preliminary analysis, we identified modifications to the previously published architecture^9^ that could contribute to the performance of our model. The two changes that were most important were: (1) to extend our training dataset with repeated ECGs recordings during the same visit of a patient, which can be viewed as a form of data augmentation; and (2) the use of an ensemble-based model.

**Figure 1:**
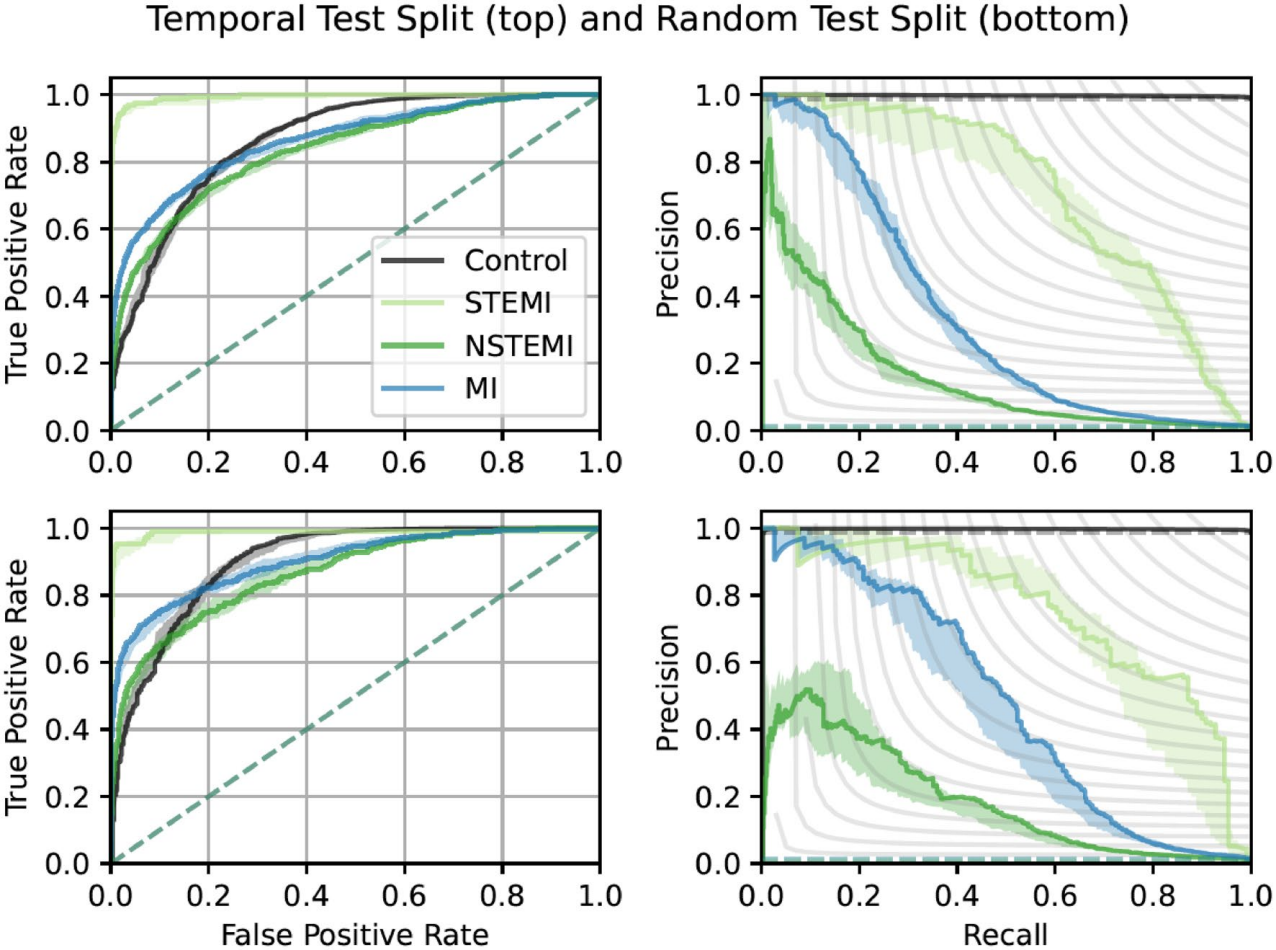
Receiver operating characteristics and precision-recall curves. Top row: Temporal test split. Bottom row: Random test split. Left column: Receiver operating characteristics curve. Right Column: Precision-recall curve. We show the curve with the median C-statistic or AP respectively as a solid line over-trained models with 10 seeds of our ensemble-based model. The shaded area shows the min and max values for all 10 models. The dashed lines indicate the curve corresponding to a random guess for the class in the given color according the legend. For the Precision-Recall curve this is a horizontal line at the value (number of positive examples)/(number of all examples). Note that the worst-case curves are overlapping for NSTEMI, STEMI and MI in the precision recall curves due to the small fraction of positives (see dashed line close to the bottom). In the Precision-Recall curves the grey curves depict iso-F1 curves. Each curve is class *vs* all, where MI specifically is STEMI and NSTEMI *vs* Control.

The performance of our model in the two test sets of the study sample (random and temporal) and a publicly available external database, PTB-XL, is described in Table 2 including model uncertainty over ten different initialization seeds. Additionally in Supplementary Table 4, we show bootstrapped data uncertainty with similar performance, details for this are in the Supplementary Methods. In the random test set, STEMIs could be discriminated with a C-statistic of 0.991 and the model had a Brier score of 0.001. For NSTEMIs, the model had a C-statistic of 0.832 and a Brier score of 0.008, with lower precision than STEMIs. The temporal test set, which contained data from emergency department visits that did not overlap in time of the development set, resulted in a C-statistic of 0.985 for STEMI and 0.867 for NSTEMI. Figure 1 illustrates receiver operating characteristics and precision-recall curves for multiple independently trained models on the two test splits of the study sample. Calibration was acceptable (calibration plots in Supplementary Fig. 3, predicted probabilities in Supplementary Fig. 4).

**Table 2:** Performance of the model in the random and temporal test set of the study sample as well as the external PTB-XL test set.

		Random	Temporal	PTB-XL
N (%)	Control	88,742 (98.9)	27,561 (98.7)	200 (72.7)
	STEMI	193 (0.2)	108 (0.4)	25 (27.3)
	NSTEMI	820 (0.9)	263 (0.9)	–
	MI	1,013 (1.1)	371 (1.3)	–
C-statistic (↑)	Control	0.863 (0.860–0.870)	0.903 (0.896–0.909)	0.962 (0.953–0.97)
	STEMI	0.991 (0.988–0.994)	0.985 (0.983–0.987)	0.932 (0.904–0.959)
	NSTEMI	0.832 (0.828–0.841)	0.867 (0.859–0.876)	–
	MI	0.863 (0.860–0.870)	0.903 (0.896–0.909)	–
AP (↑)	Control	0.998 (0.998–0.998)	0.998 (0.998–0.998)	0.955 (0.934–0.972)
	STEMI	0.692 (0.641–0.727)	0.744 (0.716–0.773)	0.954 (0.935–0.971)
	NSTEMI	0.160 (0.134–0.168)	0.184 (0.144–0.214)	–
	MI	0.330 (0.307–0.347)	0.466 (0.42–0.484)	–
Brier (↓)	Control	0.009 (0.009–0.009)	0.009 (0.009–0.010)	0.145 (0.126–0.158)
	STEMI	0.001 (0.001–0.001)	0.002 (0.002–0.002)	0.184 (0.167–0.196)
	NSTEMI	0.008 (0.008–0.008)	0.008 (0.008–0.009)	–
	Multiclass	0.018 (0.018–0.018)	0.019 (0.019–0.020)	–
ECE (↓)	Multiclass	0.417 (0.416–0.418)	0.415 (0.415–0.417)	0.277 (0.257–0.297)

Results of the model in the two study sample test sets and the publicly available PTB-XL dataset as comparison in the rightmost column. Sample size is given by each test set and outcome label together with proportion out of the given test set. Performance metrics are given as median (minimum–maximum) over ten trained models initiated with different seeds; each of the ten models is an ensemble consisting of five model members. Arrows indicate direction of better performance. We compute the metrics as class *vs.* all. Note that the class MI merges the classes STEMI and NSTEMI in one class. ECE is for multi-class calibration instead of class-wise calibration. STEMI, ST-elevation myocardial infarction; NSTEMI, non-ST-elevation myocardial infarction; AP, Average Precision or equivalently Area Under the Precision-Recall curve; ECE, Expected Calibration Error.

To ensure that our model did not use any proxies for its predictions, we stratified the test datasets according to different possible confounders. Supplementary Figs. 5 (C-statistic) illustrate results stratified according to test set records (1) without any filter applied based on ST-elevation label noise, (2) ST-elevation filter corresponding to results in main table (used in all following test set subsets); (3) stricter ST-elevation where “possible STEMI” (see Supplementary Methods) were removed from both STEMI and NSTEMI cases, (3) age tertiles, (4) sex, (5) ECG collected at the same day as the admission or not, (6) emergency department visit at Karolinska Hospital (main source of data) or another emergency department in the Stockholm region, (7) patients attending the CCU only, (8) ECGs recorded using the most common machine type (MAC55) or not, (9) ECGs recorded using the most common software (v237) or not. Apart from analyses restricting the test set controls to CCU controls only, we observed no major changes to the model performances, indicating that the predictions are not driven by these potential confounders.

As an additional test, we trained a model with only ECG traces as input, omitting age and sex. The results were almost identical to our main results indicating that the explicit addition of age and sex to the model was not crucial for our results. Furthermore, we compare our results with a model trained on data from patients at the coronary care unit alone. This dataset has 16,628 ECGs, i.e. a subset of 3.4% of our current dataset, omitting most of the control patients which were not admitted to the coronary care unit. The results from this model show that the larger dataset including all controls is important for our performance.

**Figure 2:**
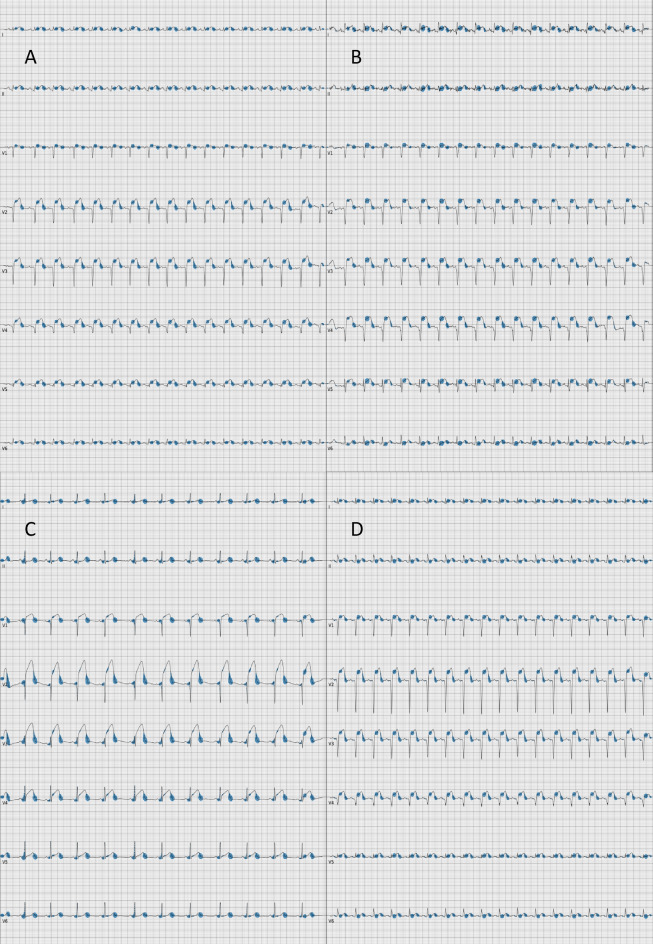
Four representative STEMIs correctly classified with high probability. Four Grad-CAM plots of STEMIs correctly classified with high probability, highlighting the parts in the ECG that the model focuses on for its prediction. Gradients corresponding to the activations in the first convolutional layer of the neural network are averaged to get the proportional importance of each channel, which is then used to compute a proportional mean of the activations. Positive values obtained were plotted as blue disks overlaid on top of the ECG, with size proportional to its magnitude.

**Figure 3:**
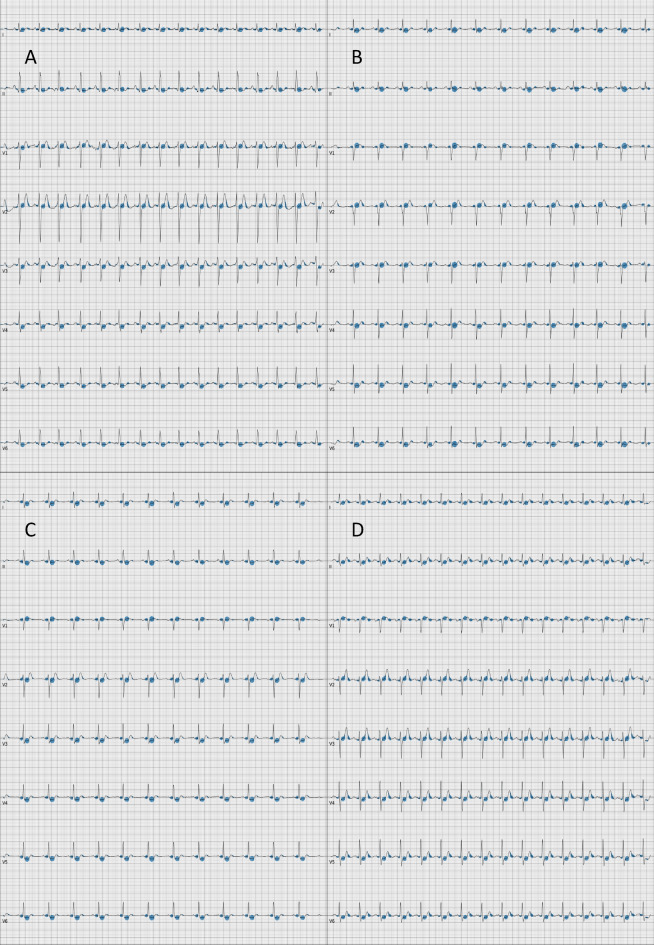
Four representative NSTEMIs correctly classified with high probability. Four Grad-CAM plots of NSTEMIs correctly classified with high probability, highlighting the parts in the ECG that the model focuses on for its prediction. Gradients corresponding to the activations in the first convolutional layer of the neural network are averaged to get the proportional importance of each channel, which is then used to compute a proportional mean of the activations. Positive values obtained were plotted as blue disks overlaid on top of the ECG, with size proportional to its magnitude.

Inspecting Grad-CAM plots yielded new insights. Figure 2 illustrates four STEMIs correctly classified with high probability. In all four panels, the model focused on the ST-segment, which is the segment trained medical staff would focus on to detect a STEMI. But the model also used the down-sloping last part of the T-wave, which is a segment that medical staff would typically not focus on when diagnosing a STEMI. Figure 3 illustrates four correctly classified NSTEMIs. In all panels, the model had focused on the ST-segment, but had also used the last part of the PQ-segment, and the last part of the T-wave, which are segments not used by medical staff to detect a NSTEMI.

Characteristics of hospitalizations with misclassified ECGs are described in Supplementary Table 3. Among the misclassified ECGs, those misclassified as STEMI more often had perimyocarditis, valvular disease or cardiomyopathy; those misclassified as NSTEMI more often had valvular or congenital heart disease, pulmonary edema, gastric ulcer or dental traits.

## Discussion

In a very large sample of all-comers to emergency departments, we developed and validated an AI model that can discriminate STEMI and NSTEMI from non-myocardial infarctions using routine 10-s ECGs.

The model achieved excellent performance for both STEMI and NSTEMIs. Doctors’ ECG interpretation is often imprecise, with a reported accuracy of 0.69 overall for practicing physicians and 0.75 for cardiologists in controlled test settings^23^, with similar numbers reported for STEMIs^24,25^. Performance of doctors outside of such standardized settings is unknown but not likely better. In the context of the model’s performance outside the study population, we do not observe a worse performance in the temporal test set when compared to the random test set. This suggests that our model works on data outside of the training data. In addition, it performs well in the discrimination of STEMIs in the external PTB-XL database test set. Unlike STEMI, diagnosis by humans of NSTEMIs from ECGs is almost by definition futile and has seen very little research.

This study is clinically important as it uses the most relevant sample possible. These consecutive all-comers represent the real-world ECG experience for emergency doctors, with ECGs—especially among the non-infarctions—that are far from the very clear specimens in managed online databases or heavily curated samples. Notably, in Sweden today and during the study period, pre-hospital ECGs are sent to coronary care units for immediate diagnosis, so the obvious STEMI cases usually bypass the emergency department and transfer straight to the coronary intervention lab upon arrival to hospital, rendering the STEMIs in the present study the less obvious cases and the walk-ins. Hence, these are cases in great need of decision support. Further, we did not exclude difficult cases, comorbidities, or previous myocardial infarctions (except for technical reasons, we removed potentially linked hospitalizations for the same myocardial infarction, and LBBBs, which cannot per se identify an acute myocardial infarction from a single ECG, but need a prior ECG for comparison).

Other studies using deep learning models have also shown good performance but lower than our model’s performance when tested in representative settings^14^, with reports of very good and similar to our model’s performance in managed settings^14,15^. Our study differs from the previous literature in that it is a multicenter study using real-world data of consecutive patients with very few exclusions, and with output labeling by many doctors. Descriptions of the clinical setting and the controls are sometimes unclear^15^. Past studies have seldom investigated NSTEMIs.

In general, it is difficult to determine which model performs best in practice since there exists no publicly available test dataset and therefore no direct comparison to other studies. However, in terms of the used models, while other studies either use a more simple convolutional network without residual connections^15^ or assume that each lead is independent by using a ResNet for each lead^14^, we do not include this assumption in our ResNet and therefore present the most general model. We tried to keep the model as standardized as possible with minimal modifications from a standard ResNet architecture to improve reproducibility.

Our model performed slightly better in younger than older patients for NSTEMI classification, and slightly better for STEMI discrimination in men than in women. Younger NSTEMI patients might have fewer underlying diseases, potentially making the prediction simpler. Men contribute to around 2/3 of the myocardial infarctions, potentially making male infarctions easier to learn given more data. A stricter filter for label noise (based on a mismatch between original labels and updated ST-elevation annotations as described in the Supplement) shows that a stricter filter improved the result for STEMI whereas the opposite was seen for NSTEMI, but the differences were minor. As expected, the more pronounced the ST-elevation was, as evaluated by a senior cardiologist, the higher was the model’s predicted probability of STEMI as seen in Supplementary Fig. 4. For NSTEMI, then difference in predicted probabilities was smaller when applying a stricter filter for label noise. Overall, the results were comparable across test set subsets and some differences in prediction results might be due to pure chance. A notable difference in the analyses restricting the test set controls to CCU controls only may be due to more underlying heart conditions in those controls compared with the large number of controls not admitted to the CCU.

The Grad-CAM plots in Figs. 2 and 3 provide important insights. The model recognizes the same ST-segment features that humans would. But the model also finds features that are novel, or that are imperceptible to the human eye. This shows an interesting way forward. We give the AI ECGs and the label, then the AI teaches us novel ways to read the ECGs. Variants of such model evaluation can likely give useful clinical and pathophysiological clues in many medical fields.

Our model’s misclassifications as STEMI follows known clinical and machine learning patterns, with perimyocarditis as an important impostor^26^. The conditions over-represented in those misclassified as NSTEMI were logical to some extent, such as valvular or congenital heart disease and pulmonary edema; the gastric ulcer and dental traits more surprising.

Some important limitations are worth mentioning. Our dataset contains some label noise. The label was determined at discharge from the coronary care unit or emergency room when the whole care episode could be summarized. The ECGs in the test sets of this study may hence not always be the ones guiding the final diagnosis. We mitigate that to some extent by using multiple ECGs if available within the day before admission in the training set, but not in the test sets. On the other hand, the hindsight allows for more stable labels for the episode as a whole, which is the ultimate goal of the classification. More information about the handling of label noise is provided in the supplementary materials. Another important limitation is the lack of an external validation sample. We did hold out the 10% of the patients with their first admission in 2016 as a temporal test set; many circumstances in that set would be similar to those in the earlier training set, but a restructuring of the Stockholm region emergency department logistics which is our main data source during the data collection period did change the composition of the sample. Furthermore, we make use of the publicly available PTB-XL dataset which does include data containing STEMI but not NSTEMI. In this dataset, our model achieves good discriminative performance. No publicly available data repositories contain ECGs with NSTEMI labels to test this externally. While the calibration of our model was better than that of comparable models, there is still room for improvement; calibration is indeed an underappreciated property in general. We did not consider transferring learned features, only model architecture, from a previous study^9^. An exploration of potential improvements in model convergence speed and final performance boost by pre-training on a different ECG classification task with a dataset in a different context may be useful, but may also introduce model biases from the other dataset. Lastly, we did not compare the performance of this ECG model to troponin-based or other methods of diagnosing myocardial infarction. While this study shows that both STEMI and NSTEMI discrimination can be performed on a general, non-curated population of emergency department patients, further research is needed for clinical use. This includes among other points, (1) the performance improvement in terms of precision for the NSTEMI class to reduce the number of false-positive predictions; (2) the improvement of the model calibration for meaningful predicted probabilities; (3) clinical usefulness should be evaluated in a randomized trial; and (4) understanding of needs of training of medical staff to work in a collaborative environment with model predictions as decision support tools. Already at this stage, we believe that this study shows the power of deep models for the general, real-world population of patients for this task. With the use of a standardized model structure from the deep learning community, we ensure that future research can be boosted from our model to obtain a clinically useful model in the near future.

In conclusion, we developed and validated a deep learning model with excellent performance in discriminating between NSTEMI, STEMI and controls on the presenting ECG of a large real-world sample of general emergency department patients. Considering the high and rising emergency department care costs and the high numbers of missed myocardial infarctions at emergency departments, our model could be of clinical value for ECG decision support, with promise of further performance development.

## Methods

### Study sample

**Figure 4:**
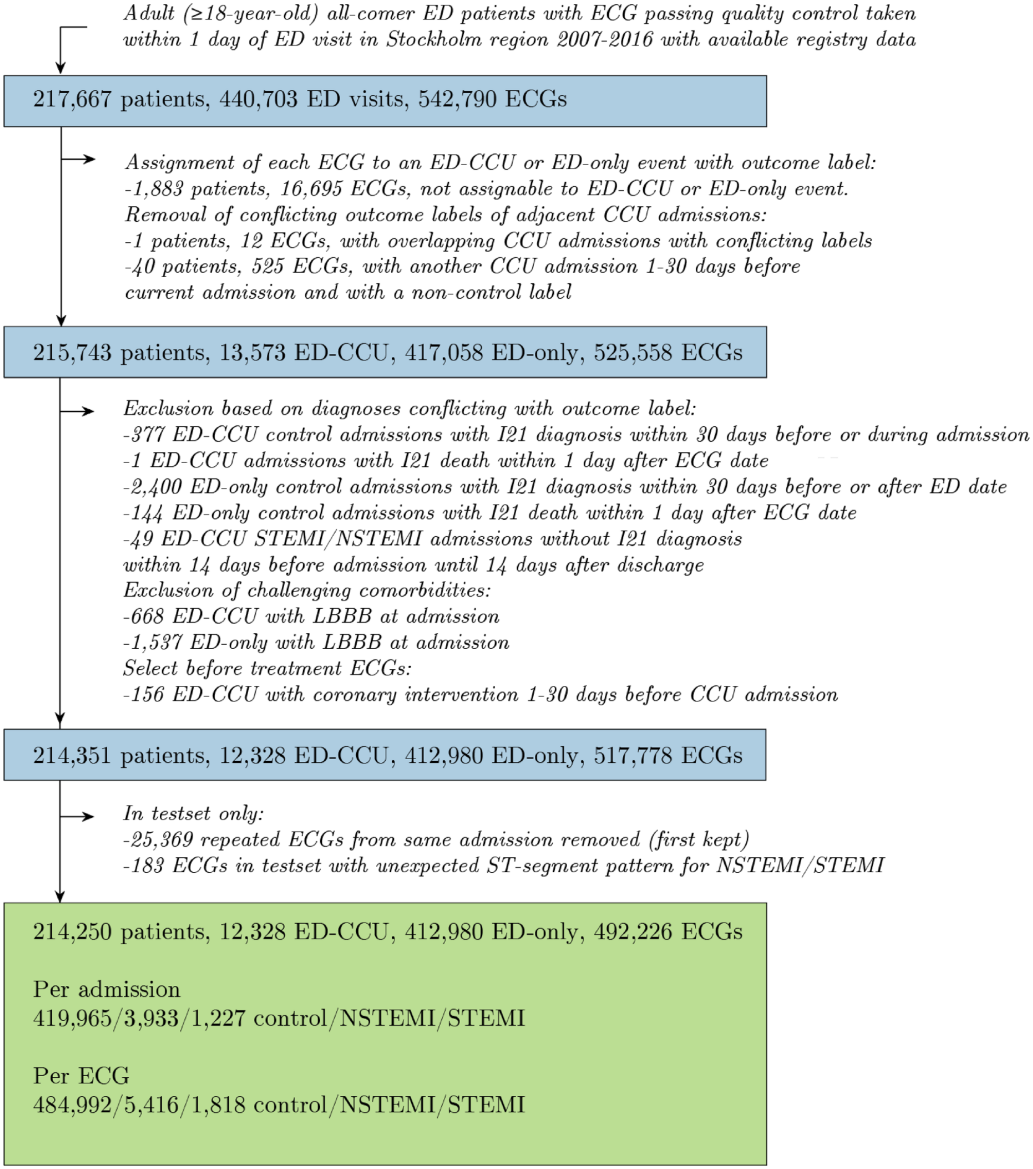
Derivation of the study sample. Inclusion/exclusion criteria applied to define the study sample. SWEDEHEART, Swedish Web-system for Enhancement and Development of Evidence-based care in Heart disease Evaluated According to Recommended Therapies; RIKS-HIA, Register of Information and Knowledge About Swedish Heart Intensive Care Admissions; CCU, coronary care unit; ED, emergency department; ED-CCU, ED visit with CCU admission with outcome label and ECG within 0–1 days prior to CCU admission and ED date within 0–1 days prior to CCU admission; ED-only, ED visit without any CCU admission within ± 30 days of ECG recording; NSTEMI, non-ST-elevation myocardial infarction; STEMI, ST-elevation myocardial infarction.

We utilized a consecutive sample of adult all-comer patients attending emergency departments in the Stockholm region between 2007 and 2016, for whom a routine ECG was obtained upon their presenting complaint. Details of the data sources are described in the Supplementary Methods. The procedure and criteria used to define the study sample are described in Fig. 4, with available predictor and outcome data described below and in Supplementary Methods. In total, 217,667 patients had at least one registered emergency department visit within the study period with at least one valid ECG recording within 1 day of visit. The emergency department visits were either followed by a coronary care unit (CCU) admission (NSTEMI/STEMI/control) or had no subsequent CCU admission (control only). After applying the sequence of filters described in Fig. 4 to ensure inclusion of at-event before-treatment ECGs, as well as confirming the outcome label, 214,250 patients with 492,226 ECGs were available for analysis, representing a total of 12,328 CCU admissions and 412,980 non-CCU visits. Out of these ECGs, 67,137 exams were repeated recordings, i.e. the same patient had multiple ECG exams in the same visit (used in training the model only). The study was approved by the Swedish Ethical Review Authority, application number 2020–01654. Informed consent was waived in this study by all applicable ethics review boards, i.e. the Region Stockholm Ethical Review Board and the Swedish Ethical Review Authority. All methods were performed in accordance with the relevant guidelines and regulations.

### Data sources

Data on the predictors and outcome of the trained model were available for all included patients from discharge records from the emergency departments, electronic health records, from linked hospitalizations, and from the SWEDEHEART registry with patient records joined on the Swedish personal identifier number of the patient. Data sources and definitions used are described in Supplementary Methods and Supplementary Table 1.a and 1.b. We only included patients with complete data on the predictors and outcome in the analyses.

### Predictors

**Figure 5:**
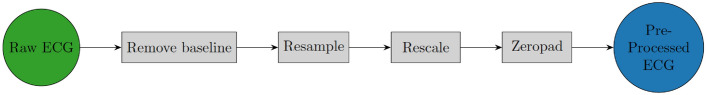
Data pre-processing of ECGs. From the raw input ECG we removed the baseline by filtering the tracing with a high-pass filter to remove biases and low-frequency trends. The filter is an elliptic filter with a cut-off frequency of 0.8 Hz and an attenuation of 40 dB, applied to the forward and reverse direction to obtain zero-phase distortion. We then resampled all ECGs to 400 Hz and zero-padded to a fixed length of 4096 samples, since the convolution-based model requires a fixed input size. For duplicated ECGs with identical data and collection time, the first copy was kept. ECGs where one or more required leads were missing or contained all-zero entries were removed. We used one-hot encoding for sex and normalized the age with the mean and standard deviation of the training dataset.

As predictors in the model, we used digital ECG data, age and sex, as in a previous study^15^, to make the model as transportable and unbiased as possible. Standard 10-s 12-lead ECG recordings sampled at 250–500 Hz were used; 8 leads were used in the present study as 4 of the standard leads are linear combinations of these 8 and are hence redundant. All digital ECGs were available in the GE MUSE (v9) XML format. The baseline was removed by filtering the tracing with a high-pass filter and all ECGs were then resampled to 400 Hz and zero-padded to a fixed length of 4096 samples. ECGs where one or more required leads were missing or contained all-zero entries were removed. Data pre-processing steps are described in more detail in Fig. 5. Age is normalized in all data to a zero-mean unit variance variable using the mean age and standard deviation from the training set and concatenated with sex as binary variable.

### Outcome

For the mutually exclusive outcome labels NSTEMI/STEMI/control status we used the high-quality SWEDEHEART registry for the CCU admissions, primarily to define NSTEMI/STEMI of the myocardial infarction cases. In addition, diagnoses from the Swedish in-patient and cause-of-death registries were used to confirm a myocardial infarction (I21) for cases, or absence of a myocardial infarction for controls (for all patients). The SWEDEHEART Riks-HIA labels which were used to define NSTEMI/STEMI are the decision of a discharging physician that followed the entire patient journey during the hospitalization; this physician had access to other exams besides the ECG, such as coronary angiograms in some patients, and blood testing in all patients. This label is the accepted gold standard for all research using SWEDEHEART data; efforts to further minimize label noise are described in Supplementary Methods.

### Training, validation, and test sets of the study sample

**Figure 6:**
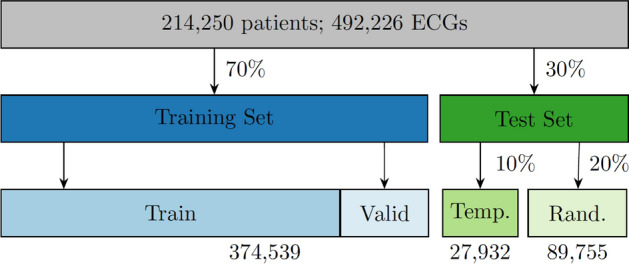
Data splitting of the study sample. The dataset was split in a training set consisting of 70% and two test sets consisting of 10% and 20% of the complete data each. The last row in the figure lists the total number of ECGs in each data split. Repeated ECG recordings at the same visit of a patient were added to the training set only.

The patients fulfilling the inclusion criteria were divided in 70%/30% splits with records from the same patient always put in the same split. The 70% split was used for training and developing the model and the 30% split, without any patient overlap, for testing the model performance. We set aside a total of 10% of the training set for validation. The 30% test split was further divided into two splits containing 20% and 10% of the study sample, to allow us to test the model in two different scenarios. The 10% split contains patients with a first recorded admission date after 2016-01-01 or later, hence temporally separated from the training data set which included patients with a first recorded admission before this date. This way the 10% test split can be used to assess the model susceptibility to trends that change with time. We denote this split the *temporal test split*. The other 20% split was sampled at random from entries with an admission date before 2016-01-01, which is the same period the 70% training split is sampled from. We denote this split the *random test split*. The process is illustrated in Fig. 6 and patient characteristics in the sets are presented in Supplementary Table 2. Our temporal test set can be considered the best possible external (temporal) validation available for the NSTEMI cases since there exists no other publicly available data set available containing ECGs and NSTEMI annotations.

### Test set of the external PTB-XL database

In addition to the study sample, an external validation dataset with 75 acute myocardial infarction cases with ST-elevation and 200 randomly selected controls was manually curated from the PTB-XL database^27,28^. The PTB-XL is a publicly available database of 21,837 10-s 12-lead ECGs annotated with 71 different ECG statements, including cases of myocardial infarction. The inclusion criteria of the PTB-XL test set used in this study is described in the Supplementary Methods.

### Model architecture

**Figure 7:**
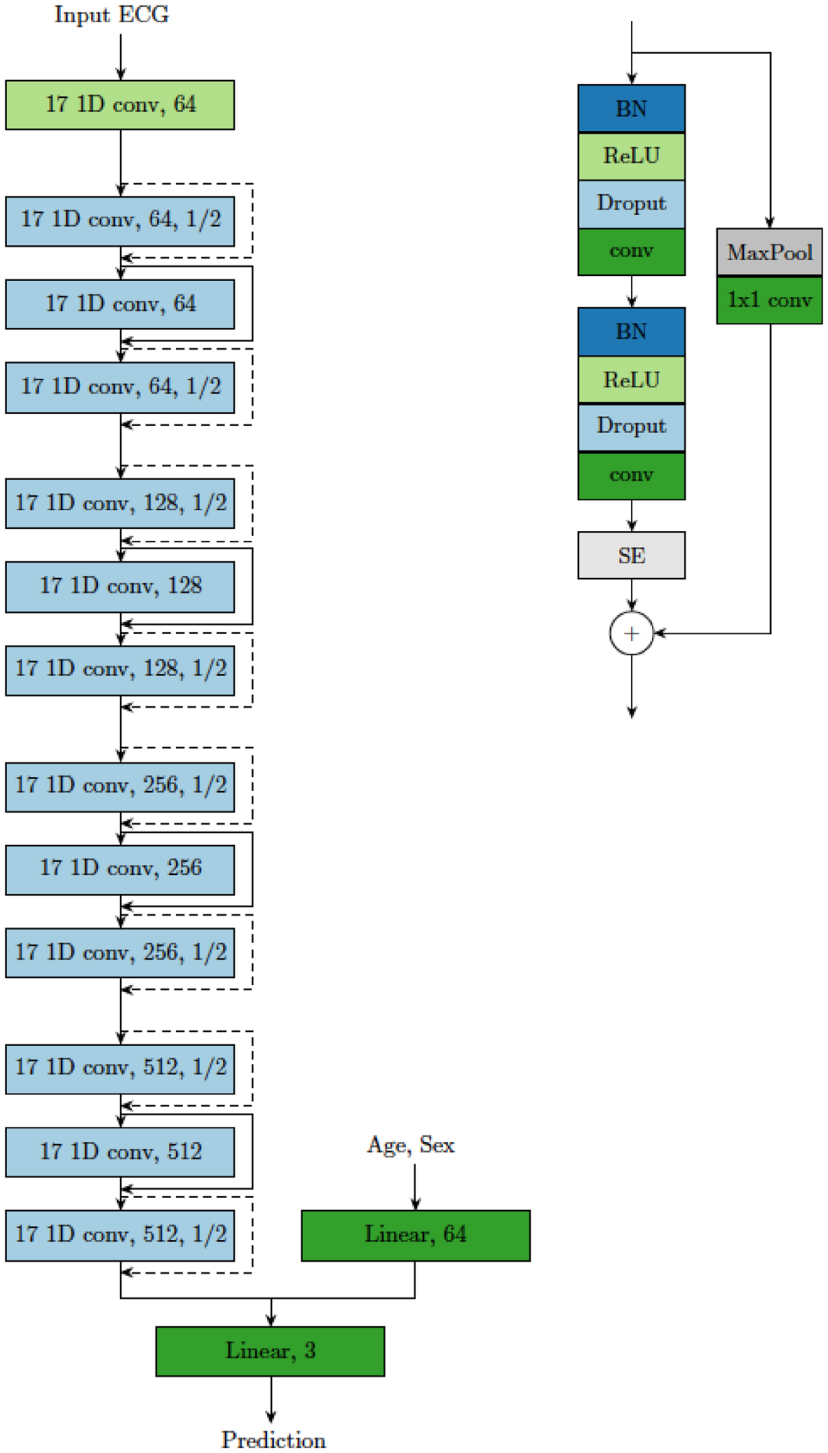
Deep neural network model architecture. The left panel is a high-level model architecture for ECG classification consisting of one part to extract features from the ECG exam and one for features from phenotypes age and sex. The light green block contains a convolutional layer followed by a batch normalization for rescaling the output and a ReLU activation function. This layer is followed by four sets of each three residual blocks in light blue, i.e. 12 residual blocks in total. The name of the block indicates the filter size of the convolutions, the number of filters and the downsampling factor (if applicable). Note that we downsample the signal by a factor of 1/2 in the beginning and ending of each set of residual blocks. The right panel illustrates the content of each residual block. Dropout is used after each nonlinear activation function as regularization. Only the first residual block does not contain the first batch normalization, ReLU and dropout layer since these layers are already applied after the initial convolution layer. We rearrange layers from the initial architecture^9^ and extend it with Squeeze and Excite (SE) blocks. This operation helps to weight the channel-wise information. Downsampling residual blocks consist in the residual skip connection (dashed lines) of a Max Pooling operation followed by a convolutional layer with filter length 1 to match the dimensions with the main branch for the summation. The remaining skip connections (full lines) do not contain any operations since input and output dimensions of the residual block are equal.

Our Deep Neural Network (DNN) model architecture is an extension of a previous model architecture^9^, for which the DNN was trained to detect six types of ECG abnormalities^29^. The full model architecture is depicted and described in Fig. 7.

In the first part of the model, to process the raw ECGs, we used a neural network based on convolutional layers similar to a residual network (ResNet) that is commonly used in image classification, but adapted here to unidimensional signals. This architecture allows DNNs to be efficiently trained by including skip connections. We adapt a modification of layer arrangements within the residual block and a skip connection which is shown to be more effective^22^.

In the second part of the model, we concatenate age with sex and pass it through a fully connected layer. The output of both model parts are flattened and concatenated to obtain one feature vector. The resulting features were used in the final linear classification layer which outputs the model prediction. The output of the trained model was the probabilities of the three mutually exclusive outcome classes NSTEMI/STEMI/control.

Ensembles of neural network models improve predictive performance^30^, and lead to better calibrated models. Therefore, we expanded our model as an ensemble of five model members. We followed the ensemble strategy of Lakshminarayanan et al.^31^ by using the same model structure and training method for each ensemble member. The only difference between the different ensemble members are the random initializations of the model parameters. This leads the model during the optimization process to reach different minima in the optimization loss landscape. Hence, the logits as outputs of the different ensemble members will be different. These logits were averaged to obtain the final prediction. A detailed description of all model hyperparameters is provided in the Supplementary Methods.

### Model training and validation

The model was trained by minimizing the cross-entropy loss for 100 epochs. Details about the training hyperparameter and regularization terms are described in the Supplementary Methods. In the training dataset, we made use of the repeated ECG exams of patients who had multiple exams at an emergency department visit, but not for validation and testing. We consider this as a form of data augmentation since these exams have the same label but are recorded at different times and therefore with a slightly different state of the patient and possibly different placement of the ECG leads. For validation and testing, only the first recorded ECG of an admission was used. Details are in the Supplementary Methods. Note that we improve the training procedure and model architecture from a previous study^9^ with established methods^32^. The details for the model and training improvements as well as all hyperparameters for model training such as learning rate and regularization parameters are described in Supplementary Methods.

### Hyperparameter search

We conducted a search over 6 hyperparameters and their respective values. This gives a total of 2025 possible combinations. Due to the high number of possible combinations, we ran a random search for 345 combinations. The remaining model and training hyperparameters were fixed in this search since they have been proven efficient in a previous study with a model architecture which the present study extends^9^. We evaluated the tested hyperparameters in the validation dataset. Note that during the search we only train a single model and not an ensemble of multiple models. Details of the hyperparameter search and its related computational cost are outlined in the Supplementary Methods.

### Model discrimination and calibration

Performance of prediction models is often quantified using the C-statistic that measures discrimination, which is also used in the present study, i.e. the model’s ability to assign higher risk estimates/probabilities to patients who will experience the event than patients who will not. While discrimination is important, it tells us nothing about the reliability of the probability estimates. Calibration, i.e. if the model’s probability estimates reflect the ground truth empirical class frequencies, is a more important property of the model for clinical use. One of our main concerns was model calibration, since modern DNN architectures are generally poorly calibrated^33^. As metrics of model calibration, we focused on the Expected Calibration Error (ECE) and the Brier score. The ECE is the weighted absolute difference between the class membership and the estimated probability for that class averaged over 15 bins, while the Brier score measures the average squared error on the probability scale. Both metrics are applicable to both binary and multiclass problems. We visualized the calibration of our model using calibration plots, also called reliability diagrams.

### Evaluation of correct versus incorrect high-probability classifications

We investigated possible patterns for our models correct and incorrect classifications of STEMIs and NSTEMIs. First, we used Grad-CAM plots to highlight the parts of the ECG that the model focuses on—meaning the sections of the input ECG where the model puts its most weight on—to make its predictions^34^. This method generates the visualization in two steps: In a forward pass step, it computes the activations of the neural network in a given intermediary layer (in our case, the first convolutional layer). And in a backward step, it computes the gradients corresponding to these activations. The gradients are averaged to get the proportional importance of each channel, which is then used to compute a proportional mean of the activations. Positive values obtained were plotted as purple disks overlaid on top of the ECG, with size proportional to its magnitude, to generate the visualization. One senior cardiologist (JS) inspected the Grad-CAM plots from the ten cases with the highest estimated probability for each myocardial infarction class and selected four illustrative plots per class.

We tested the over/underrepresentation of inpatient and specialized outpatient care diagnoses at the time of the visit among correctly classified *versus* misclassified patients with a predicted probability > 0.5 in pairwise independent tests to find underlying diagnoses where the model often made incorrect classifications.

## Supplementary Information

Supplementary Information.

## Data Availability

The data that support the findings of this study are available from the Swedish Board of Health and Welfare and the included healthcare regions, but restrictions apply to the availability of these data, which were used under license for the current study, and so are not publicly available. Data are however available from corresponding author (J.S.) upon reasonable request and with permission of the Swedish Board of Health and Welfare and the included healthcare regions. All code together with the parameter/hyperparameter estimates of the presented models is available upon request.
