# Supplementary material for: Development and validation of deep learning ECG-based prediction of myocardial infarction in emergency department patients
Source: Sci Rep. 2022 Nov 15;12:19615. doi: 10.1038/s41598-022-24254-x (PMC9666471; doi:10.1038/s41598-022-24254-x)
Supplement: Supplementary file 1 — Supplementary Information. [file 41598_2022_24254_MOESM1_ESM.docx]

Development and validation of deep learning ECG-based prediction of myocardial infarction in emergency department patients

# Supplementary Methods

## Data sources

Adult patients (>= 18 years old) with available emergency department data from 6 emergency departments in the Stockholm region, Sweden, between 2003 and 2017 were collected. The sample was linked to national registries (the patient [inpatient and specialized outpatient], prescribed drug, and death registries), national quality registries (SWEDEHEART [Swedish Web-system for Enhancement and Development of Evidence-based care in Heart disease Evaluated According to Recommended Therapies; a Swedish nation-wide quality register] sub-registries RIKS-HIA [Register of Information and Knowledge About Swedish Heart Intensive Care Admissions] and SCAAR [Swedish Coronary Angiography and Angioplasty Registry]), as well as a regional database of ECGs (Karolinska ECG database) and electronic health records as summarized in **Supplementary Table 1.a**. All data sources covered the time-period 2007-2016 or longer. Characteristics have been described for STEMI^1^ and NSTEMI^2^ patients in SWEDEHEART during the present study period, and the study sample has been partially described previously.^3^ All ECG recordings were available as exported XML files in the GE MUSE (v9) XML format, with each lead represented by a numerical signal vector together with meta-data of the recording.

The outcome label used, NSTEMI/STEMI/control, is the standard INFARCTTYPE variable in SWEDEHEART RIKS-HIA,^4^ complemented by diagnoses from the Swedish in-patient and cause-of-death registries to confirm a myocardial infarction (I21) for cases, or absence of a myocardial infarction for controls.

The SWEDEHEART variable captures the view of the whole cycle of care by the attending cardiologist at time of discharge from the coronary care unit, who has access to all relevant patient data, including but not limited to single ECGs, continuous ECG monitoring, cardiac enzyme series and other lab data, and angiographic and echocardiographic results. Regular monitoring of the SWEDEHEART registry shows a data accuracy of around 96%.^5^

## Label Noise

We ran a risk of label noise since the label (control/STEMI/NSTEMI) was determined at discharge from the hospital, when the whole care episode, which can include multiple ECGs, could be summarized. Hence, it is impossible for us to identify which ECG determined the final diagnosis of the discharging physician. We try to include the correct ECGs by inclusion filters at-event before-treatment. However, in the end the ECG data of this study may not always be the ones guiding the final diagnosis. We mitigate that to some extent by using multiple ECGs, i.e. the repeated recordings if available within one day before or on admission in the training set, but not in the validation and test sets.

We evaluated the label noise by checking for possible/definite ST-elevation in ECGs with the original label STEMI or for the absence of definite ST-elevation in the ECGs with the label NSTEMI or control. All those ECGs were manually evaluated by a senior cardiologist (JS) who was blinded to the original labels. Given the large number of control ECGs, JS only manually evaluated 100 control ECGs selected at random and found no ECG with a possible/probable ST-elevation. All ECGs with the label STEMI/NSTEMI in the test set where checked and we found a fair amount of label noise. This does not necessarily imply that the data is wrongly labelled but that the ECG that we use based on our inclusion filters does not clearly show the characteristics for the label whereas another exam in the same hospitalization may show the characteristics.

To have a clear test set for which we report the model evaluation metrics, ECGs with a likely incorrect original label were removed. The manually evaluated ECGs were labelled as "definitely STEMI (0)", "likely STEMI (1)", "not STEMI (2)". If the original label was STEMI and our review's label was (2) we removed the ECG from the test set. Similarly, if the original label was NSTEMI and our review's label was (0), we removed the ECG. In this way we removed 117 (28%) STEMI and 66 (6%) NSTEMI ECGs from the test data set.

## Model architecture

Our model architecture is an extension of a previous study.^6^ We extended the architecture with Squeeze and Excite (SE) blocks^7^ within each residual block. Additionally, we include the phenotypes age and sex in our model. Our final model has the following hyperparameter. We use 12 residual blocks with filter sizes {64;64;64;128;128;128;256;256;256;512;512;512} and down sampling factors {2;1;2;2;1;2;2;1;2;2;1;2} where all SE layers have a reduction factor of 8. The convolutional layers have a kernel size of 17. Age is normalized in all data to a zero-mean unit variance variable using the mean age and standard deviation from the training set and concatenated with sex as binary variable. The combined age and sex is then encoded in a linear layer with 64 output units before it gets concatenated with the flattened ResNet output. In the end we create an ensemble of 5 models which are independently trained from scratch.

## Model training

The model was trained by minimizing the cross-entropy loss using the Adam optimizer with default parameters and learning rate of 0.0005 for 100 epochs using an effective batch size of 1024 distributed over two GPUs. We used a cosine learning rate scheduler which reduces the learning rate according to a cosine function from the initial learning rate to a final value of 10^-6^ over the epochs. Initially, we warmed up the learning rate linearly over 10 epochs. For regularization we use dropout with dropout probability of 0.5 within the ResNet blocks. Furthermore, we regularized with weight decay of 10^-3^. We tried label smoothing for additional regularization during the hyperparameter search but found an optimal value of 0.00.

## Model and training improvements

We extended the training procedure with multiple training and architecture options following previous recommendations of modern ResNet tuning to improve model performance.^8, 9^ We tested to include these options iteratively on a subset of the training data set with high-risk patients who were admitted to a coronary care unit, with increased number of training epochs up to 200 using 5-fold cross validation.

1. Training data set augmentation: include the repeated recordings in the train data set.
2. Learning rate scheduler: move from an initial multistep learning rate scheduler with fixed decrease by factor 10 at epoch 75, 125, 175 to a cosine learning rate scheduler with linear warmup.^10^
3. Label smoothing.
4. Additional SE net layers.
5. Additional age and sex embedding.
6. Additional ensemble-based model with heuristically chosen five ensemble members.

Our observation was that each of the extensions improves our evaluation metrics. Therefore, our model for the hyperparameter search contains all of the extensions. Furthermore, we experimented with the SGD optimizer instead of ADAM without and with momentum of {0.7, 0.8, 0.9} and with increase of the network width by factors of {1, 1.5, 2}. However, these results did not improve our performance.

## Hyperparameter Search

For our random hyperparameter search we considered the following model hyperparameter and respective values: number of residual blocks {4, 8 ,12} with respective filter sizes and down sample factors, reduction factor of the SE-layer {4, 8, 16}. For the training hyperparameters we considered the following: batch size per GPU {256, 512, 1024}, learning rate {0.05, 0.01, 0.005, 0.001, 0.0005}, weight decay {10^-3^, 10^-4^, 0}, loss smoothing {0, 0.05, 0.1, 0.15, 0.2}.

We ran our model training on 2 Nvidia A100-PCIE-40Gb GPUs. We utilize distributed data parallel training with multiple parallel spawned processes. This effectively doubles our batch size and therefore increases training speed significantly. In this setup training of one model - not five models for the ensemble - takes around 4 hours for 100 epochs.

## Model calibration

We tried to improve upon the original calibration by temperature scaling,^11^ vector calibration,^11^ and Dirichlet calibration.^12^ None of those methods succeeded in improving the model calibration. Further work is necessary to investigate the possibility to improve model calibration beyond our current setting.

## External validation set

The PTB-XL is a publicly available database of 21,837 10-second 12-lead ECGs annotated with 71 different ECG statements, including cases of myocardial infarction.^13, 14^

- 98 ECGs annotated as likely acute myocardial infarctions without electrode problems by at least one cardiologist where selected based on the following PTB-XL annotations (infarction_stadium1==”Stadium I” OR infarction_stadium2==”Stadium I”) AND validated_by_human==TRUE AND isna(electrodes_problem).
- 200 ECGs annotated as likely controls without myocardial infarction and without electrode problems by at least one cardiologist where selected based on the following PTB-XL annotations (isna(infarction_stadium1) AND isna(infarction_stadium2)) AND validated_by_human==TRUE AND isna(electrodes_problem) AND NORM==100.

These ECGs were manually reviewed by a senior cardiologist (JS). All controls were confirmed and 75 myocardial infarction ECGs had a probable or definite ST-elevation in line with the existing PTB-XL annotation, which were included in the PTB-XL test set. The leads used in training were extracted and normalized as described in Figure 3. Age and sex was extracted from the PTB-XL database and age was normalized using the mean and standard deviation from the training dataset.

## Bootstrapped data uncertainty

In **Table 2** we report model uncertainty from different initialization of our convolutional network. In **Supplementary Figure 4** we additionally report data uncertainty based on bootstrapped data from the test data sets. For the bootstrap we chose the model with the median micro average precision on the validation data. Since we have 10 models, we cannot chose the median but chose the 6^th^ best model in terms of micro average precision. Then we make 1000 samplings with replacement from each respective test data set and report the mean and 95% confidence interval (percentile bootstrap) for all metrics.

# Supplementary Tables

**Supplementary Table 1.a.** Data sources used to define the study sample with patient records linked on the Swedish personal identifier number.

| **Database** | **Coverage** | **Type of data** |
| --- | --- | --- |
| Emergency department discharge records | Regional | Presented complaint |
| Electronic health records | Regional | ECGs, laboratory measurements |
| SWEDEHEART Riks-HIA | National | CCU admissions with STEMI/NSTEMI labels |
| SWEDEHEART SCAAR | National | Coronary interventions |
| National Patient Registry | National | Diagnoses, surgical interventions |
| Cause of death registry | National | Causes of death |
| Swedish prescribed drug registry | National | Dispensed drugs |

**Supplementary Table 1.b.** Definitions used for diagnoses, surgery, and pharmaceutical treatment.

| Diagnosis/intervention/treatment | Codes |
| --- | --- |
| *Diagnosis (ICD10)* | |
| Myocardial infarction | I21 |
| Unstable angina | I20.0 |
| Ischemic heart disease | I20-I25 |
| Left bundle branch block | I44.(6\|7) |
| Stroke | I60-I64 |
| Peripheral artery disease | I70-I74,I77.(3\|6\|8),I79 |
| Heart failure | I50 |
| Atrial fibrillation | I48 |
| Cardiovascular disease | I |
| *Surgical codes (KVÅ)* | |
| PCI/CABG | FNG(00\|02\|05\|10\|96),FNC,FND,FNE |
| *Treatment (ATC)* | |
| Renin-angiotensin system inhibitors | C09 |
| Calcium channel blockers | C08 |
| Beta-receptor blockers | C07 |
| Mineralocorticoid receptor antagonists | C03DA |
| Diuretics | C03 |
| Anti-arrhythmic drugs | C01B |
| Statins | C10AA |
| Anticoagulants | B01A(A\|E\|F) |
| Antiplatelets | B01AC |

**Supplementary Table 2.** Clinical characteristics of the study sample, stratified by control/NSTEMI/STEMI and training/test set.

|  | Random test sets | | | Temporal test sets | | | Training sets | | |
| --- | --- | --- | --- | --- | --- | --- | --- | --- | --- |
|  | **Control** | **NSTEMI** | **STEMI** | **Control** | **NSTEMI** | **STEMI** | **Control** | **NSTEMI** | **STEMI** |
| Number of patients | 88,742 | 820 | 193 | 27,561 | 263 | 108 | 368,689 | 4,333 | 1,517 |
| *Clinical characteristics at ED visit* | | | | | | | | | |
| Age | 64.0  (47.0,77.0) | 71.0  (61.0,80.2) | 66.0  (60.0,77.0) | 54.0  (37.0,71.0) | 70.0  (59.5,79.5) | 64.0  (55.0,71.0) | 65.0  (48.0,78.0) | 72.0  (62.0,81.0) | 67.0  (57.0,78.0) |
| Male | 47.2 | 67.8 | 75.1 | 48.1 | 66.5 | 80.6 | 47.2 | 64.9 | 73.0 |
| Year | 2012  (2010, 2014) | 2013  (2011, 2015) | 2013  (2011, 2014) | 2016  (2016, 2016) | 2016  (2016, 2016) | 2016  (2016, 2016) | 2012  (2010, 2014) | 2013  (2011, 2015) | 2013  (2011, 2014) |
| *Presenting complaint* | | | | | | | | | |
| Chest pain | 21.5 | 71.5 | 67.4 | 22.3 | 74.1 | 80.6 | 21.4 | 71.1 | 69.7 |
| Difficulty breathing | 14.1 | 13.4 | 7.3 | 12.5 | 10.6 | 3.7 | 14.8 | 12.2 | 5.9 |
| Dizziness | 7.6 | 0.4 | 2.1 | 9.4 | 0.0 | 0.9 | 6.8 | 0.9 | 1.1 |
| Heart problems | 2.0 | 1.5 | 3.6 | 0.0 | 0.0 | 0.0 | 2.2 | 1.3 | 1.8 |
| Circulatory arrest | 0.1 | 0.7 | 5.2 | 0.2 | 1.5 | 6.5 | 0.1 | 1.1 | 3.3 |
| *Cardiovascular diagnoses prior to ED visit** | | | | | | | | | |
| Myo-cardial infarction | 8.5 | 24.5 | 12.4 | 3.3 | 14.4 | 9.3 | 8.8 | 27.0 | 17.1 |
| Unstable angina | 3.9 | 11.5 | 3.1 | 1.3 | 5.3 | 1.9 | 4.5 | 11.5 | 7.2 |
| Ischemic heart disease | 20.5 | 40.4 | 19.2 | 7.7 | 27.0 | 14.8 | 21.0 | 44.7 | 25.0 |
| Stroke | 9.3 | 11.5 | 5.2 | 3.8 | 3.0 | 3.7 | 9.4 | 11.6 | 7.9 |
| Peripheral artery disease | 7.0 | 12.2 | 5.7 | 3.6 | 5.7 | 7.4 | 7.2 | 12.8 | 7.3 |
| Heart failure | 15.6 | 20.4 | 5.7 | 4.8 | 9.9 | 2.8 | 16.7 | 21.3 | 10.6 |
| Atrial fibrillation | 19.0 | 16.3 | 5.2 | 7.7 | 9.9 | 0.9 | 21.1 | 16.1 | 9.8 |
| Cardio-vascular disease | 58.7 | 69.6 | 43.5 | 37.5 | 54.0 | 41.7 | 60.6 | 72.0 | 54.1 |
| *Drugs with >=1 dispensation within one year prior to ED visit* | | | | | | | | | |
| Renin-angio-tensin system inhibitors | 32.7 | 51.8 | 33.7 | 23.9 | 51.0 | 30.6 | 33.7 | 51.8 | 37.7 |
| Calcium channel blockers | 17.4 | 28.7 | 23.3 | 13.0 | 27.0 | 13.9 | 18.3 | 29.5 | 21.3 |
| Beta-receptor blockers | 36.0 | 52.7 | 31.6 | 20.1 | 36.5 | 20.4 | 37.5 | 51.1 | 35.2 |
| Mineralocorticoid receptor antagonists | 6.3 | 7.7 | 4.1 | 2.1 | 3.4 | 0.9 | 6.6 | 6.0 | 3.0 |
| Diuretics | 27.4 | 36.1 | 21.2 | 12.3 | 23.6 | 5.6 | 28.8 | 34.5 | 20.1 |
| Anti-arrhythmic drugs | 1.6 | 0.5 | 0.0 | 0.4 | 0.4 | 0.0 | 1.8 | 0.4 | 0.7 |
| Statins | 23.9 | 42.2 | 20.2 | 15.4 | 29.3 | 24.1 | 24.2 | 42.6 | 24.9 |
| Anticoagulants | 12.4 | 10.6 | 1.0 | 6.8 | 4.9 | 1.9 | 13.3 | 8.7 | 6.4 |
| Antiplatelets | 27.9 | 49.0 | 26.9 | 13.4 | 30.8 | 16.7 | 28.9 | 49.8 | 30.1 |
| *Cardiac enzymes within ED visit or coronary care unit hospitalization*** | | | | | | | | | |
| Troponin I measured | 1.0 | 8.2 | 14.0 | 0.0 | 0.0 | 0.0 | 1.2 | 8.7 | 14.7 |
| Max troponin I (ng/L) | 29.7  (29.7,40.0) | 2200.0  (550,6450) | 21000.0  (5100,48850) | - | - | - | 29.7  (29.7,40.0) | 2950.0  (630,10500) | 18100.0  (2900,45000) |
| Troponin T measured | 36.4 | 86.3 | 86.0 | 37.5 | 98.9 | 100.0 | 38.7 | 86.7 | 86.9 |
| Max troponin T (ng/L) | 9.9  (5.0,16.0) | 228.5  (80.8,777.8) | 2315.0  (684,5720) | 5.0  (5.0,12.0) | 231.5  (103.5,740.8) | 2910.0  (1205,6275) | 9.9  (5.0,20.0) | 271.0  (96.0,830.0) | 1790.0  (474.5,4500) |
| NTproBNP measured | 7.8 | 20.1 | 12.4 | 6.9 | 24.0 | 26.9 | 9.1 | 21.6 | 17.0 |
| Max NTproBNP (ng/L) | 1230.0  (269,4187.5) | 2910.0  (965,7770) | 4150.0  (349.2,7630) | 746.0  (130,2935) | 1180.0  (348.5,6130) | 1670.0  (610,4410) | 1400.0  (303,4410) | 3020.0  (802,9150) | 3300.0  (900.5,9300) |
| *Main cause of coronary care unit hospitalization**** | | | | | | | | | |
| Myocardial infarction | 0.0 | 93.4 | 95.9 | 0.0 | 96.6 | 98.1 | 0.0 | 94.0 | 97.4 |
| Unstable angina | 0.2 | 4.1 | 0.5 | 0.2 | 10.6 | 0.0 | 0.2 | 3.7 | 1.8 |
| Ischemic heart disease | 1.2 | 93.8 | 95.9 | 0.6 | 96.6 | 98.1 | 1.4 | 94.6 | 97.4 |
| Stroke | 2.2 | 0.6 | 0.5 | 1.9 | 0.8 | 0.9 | 2.0 | 1.2 | 0.7 |
| Peripheral artery disease | 0.4 | 0.2 | 0.0 | 0.4 | 0.4 | 0.0 | 0.5 | 0.3 | 0.3 |
| Heart failure | 3.1 | 2.2 | 1.0 | 1.4 | 3.0 | 0.9 | 3.5 | 2.1 | 0.9 |
| Atrial fibrillation | 4.9 | 1.2 | 0.0 | 3.0 | 0.4 | 0.0 | 6.4 | 0.8 | 0.4 |
| Cardiovascular disease | 16.8 | 95.5 | 99.0 | 12.1 | 97.7 | 100.0 | 19.3 | 96.3 | 98.2 |
| *Mortality after coronary care unit admission* | | | | | | | | | |
| 30-day all-cause death | 3.3 | 6.1 | 13.5 | 2.5 | 4.9 | 7.4 | 3.6 | 6.5 | 10.5 |
| In-hospital all-cause death | 2.4 | 5.2 | 12.4 | 1.9 | 3.8 | 5.6 | 2.7 | 5.6 | 9.0 |

Patient characteristics of the study sample, by control/NSTEMI/STEMI status and by training and test sets. Data presented as median (interquartile range) or percentages.

*Prevalent disease based on any diagnosis position, inpatient and outpatient specialist care combined.

**Combining troponin and high-sensitive troponin laboratory measurements from regional laboratory databases and the SWEDEHEART database. Maximum of all available measurements within the time window is reported separately for troponin I and T.

***Primary diagnosis from inpatient specialist care and/or specialized outpatient care at time of the ED/CCU visit.

ED, emergency department; NTproBNP, N-terminal pro-B-type natriuretic peptide.

**Supplementary Table 3.** Over-/underrepresented diagnoses among misclassified cases.

| ICD10 code | Odds ratio | Z-score | Observed class | Wrong predicted class |
| --- | --- | --- | --- | --- |
| K04 | 884.9 | 27.5 | Control | NSTEMI |
| K27 | 601.6 | 22.7 | Control | NSTEMI |
| J95 | 417.9 | 18.9 | Control | NSTEMI |
| I05 | 301.3 | 16.2 | Control | NSTEMI |
| I33 | 209.6 | 13.5 | Control | NSTEMI |
| I40 | 195.5 | 27.8 | Control | STEMI |
| I07 | 181.7 | 12.9 | Control | STEMI |
| I51 | 126.0 | 15.0 | Control | STEMI |
| J81 | 109.9 | 9.8 | Control | NSTEMI |
| Q20 | 78.8 | 8.2 | Control | NSTEMI |
| K26 | 59.9 | 7.2 | Control | NSTEMI |
| I34 | 54.9 | 6.8 | Control | NSTEMI |
| K25 | 44.7 | 6.2 | Control | NSTEMI |
| I46 | 43.2 | 10.5 | Control | STEMI |
| B95 | 37.6 | 5.6 | Control | NSTEMI |
| K62 | 33.1 | 5.5 | Control | STEMI |
| I71 | 31.8 | 5.1 | Control | NSTEMI |
| I42 | 29.5 | 8.6 | Control | STEMI |
| I47 | 26.3 | 4.6 | Control | NSTEMI |
| I30 | 24.3 | 4.6 | Control | STEMI |
| K92 | 23.9 | 4.4 | Control | NSTEMI |
| E10 | 23.3 | 4.4 | Control | NSTEMI |
| A04 | 21.9 | 4.4 | Control | STEMI |
| A49 | 21.1 | 4.3 | Control | STEMI |
| Z72 | 19.1 | 5.6 | Control | STEMI |
| N18 | 12.9 | 4.1 | Control | NSTEMI |
| I20 | 11.5 | 3.9 | Control | NSTEMI |
| Z86 | 10.0 | 4.7 | Control | STEMI |
| T81 | 0.1 | -4.0 | NSTEMI | Control |
| N17 | 0.1 | -3.9 | NSTEMI | Control |
| J09 | 0.02 | -4.7 | NSTEMI | Control |
| N13 | 0.02 | -4.7 | NSTEMI | Control |

Over-/underrepresented diagnoses when comparing correct classifications with misclassifications for a given observed class, restricted to ECGs with a high predicted probability (Pr>0.5). Tested in an asymptotic general independence test with a two-sided alternative hypothesis.^15^ All diagnoses at time of the visit (coronary care unit or emergency department only) are included (all diagnosis positions). All results with a false discovery rate (Benjamini-Hochberg) < 0.01 are reported.

**Supplementary Table 4.** Performance of the model showing bootstrapped data uncertainty

|  |  | **Random** | **Temporal** | **PTB-XL** |
| --- | --- | --- | --- | --- |
| C-statistic (↑) | Control | 0.867 (0.854-0.880) | 0.902 (0.884-0.921) | 0.958 (0.920-0.984) |
|  | STEMI | 0.994 (0.990-0.997) | 0.987 (0.963-0.998) | 0.959 (0.921-0.985) |
|  | NSTEMI | 0.837 (0.823-0.852) | 0.866 (0.842-0.889) | - |
|  | MI | 0.867 (0.854-0.880) | 0.902 (0.884-0.921) | - |
| AP (↑) | Control | 0.998 (0.998-0.998) | 0.998 (0.997-0.999) | 0.967 (0.920-0.994) |
|  | STEMI | 0.714 (0.653-0.770) | 0.757 (0.674-0.834) | 0.939 (0.898-0.971) |
|  | NSTEMI | 0.160 (0.133-0.189) | 0.191 (0.147-0.240) | - |
|  | MI | 0.343 (0.314-0.372) | 0.483 (0.428-0.534) | - |
| Brier (↓) | Control | 0.009 (0.008-0.009) | 0.009 (0.008-0.010) | 0.150 (0.114-0.189) |
|  | STEMI | 0.001 (0.001-0.001) | 0.002 (0.001-0.002) | 0.189 (0.147-0.232) |
|  | NSTEMI | 0.008 (0.008-0.009) | 0.008 (0.007-0.010) | - |
|  | Multiclass | 0.018 (0.017-0.019) | 0.02 (0.017-0.022) | - |
| ECE (↓) | Multiclass | 0.417 (0.416-0.418) | 0.416 (0.415-0.417) | 0.281 (0.240-0.324) |

Results of the model in the two test sets of the study sample and the publicly available PTB-XL dataset as comparison in the rightmost column. We show the mean and 95% confidence interval from a bootstrap to highlight data uncertainty which is in contrast to Table 2 where we show model uncertainty using different seeds.

**Supplementary Table 5.** Number and proportion of records in the random and temporal test set restricted to the strata shown in **Supplementary Figure 5**.

| Subset | Random | Temporal |
| --- | --- | --- |
| No ST-filter | 88,742 (98.7);273 (0.3);870 (1.0) | 27,561 (98.5);145 (0.5);279 (1.0) |
| Moderate ST-filter* | 88,742 (98.9);193 (0.2);820 (0.9) | 27,561 (98.7);108 (0.4);263 (0.9) |
| Strict ST-filter | 88,742 (99.1);152 (0.2);698 (0.8) | 27,561 (98.9);82 (0.3);237 (0.9) |
| Age < 54 | 29,706 (99.6);23 (0.1);87 (0.3) | 13,420 (99.6);25 (0.2);33 (0.2) |
| Age [54,73) | 30,399 (98.4);106 (0.3);382 (1.2) | 8,553 (97.8);61 (0.7);127 (1.5) |
| Age > 73 | 28,637 (98.6);64 (0.2);351 (1.2) | 5,588 (97.8);22 (0.4);103 (1.8) |
| Male | 41,913 (98.4);145 (0.3);556 (1.3) | 13,261 (98.1);87 (0.6);175 (1.3) |
| Female | 46,829 (99.3);48 (0.1);264 (0.6) | 14,300 (99.2);21 (0.1);88 (0.6) |
| ECG same day | 86,171 (99.0);182 (0.2);674 (0.8) | 26,800 (98.8);105 (0.4);228 (0.8) |
| ECG 1d apart | 2,571 (94.2);11 (0.4);146 (5.4) | 761 (95.2);3 (0.4);35 (4.4) |
| Karolinska ED | 74,061 (99.0);141 (0.2);617 (0.8) | 19,701 (98.9);61 (0.3);153 (0.8) |
| Not Karolinska ED | 14,681 (98.3);52 (0.3);203 (1.4) | 7,860 (98.0);47 (0.6);110 (1.4) |
| CCU only | 1,551 (60.5);193 (7.5);820 (32.0) | 170 (31.4);108 (20.0);263 (48.6) |
| MAC55 | 69,916 (98.8);159 (0.2);690 (1.0) | 22,848 (98.6);92 (0.4);229 (1.0) |
| Not MAC55 | 18,826 (99.1);34 (0.2);130 (0.7) | 4,713 (99.0);16 (0.3);34 (0.7) |
| v237 | 65,021 (99.0);138 (0.2);529 (0.8) | 14,530 (98.7);63 (0.4);128 (0.9) |
| Not v237 | 23,721 (98.6);55 (0.2);291 (1.2) | 13,031 (98.6);45 (0.3);135 (1.0) |

The numbers are presented in the order controls, STEMI, NSTEMI.

# Supplementary Figures

**Supplementary Figure 1.** Distributions of age at time of the ECG recording, by control/NSTEMI/STEMI status.

##
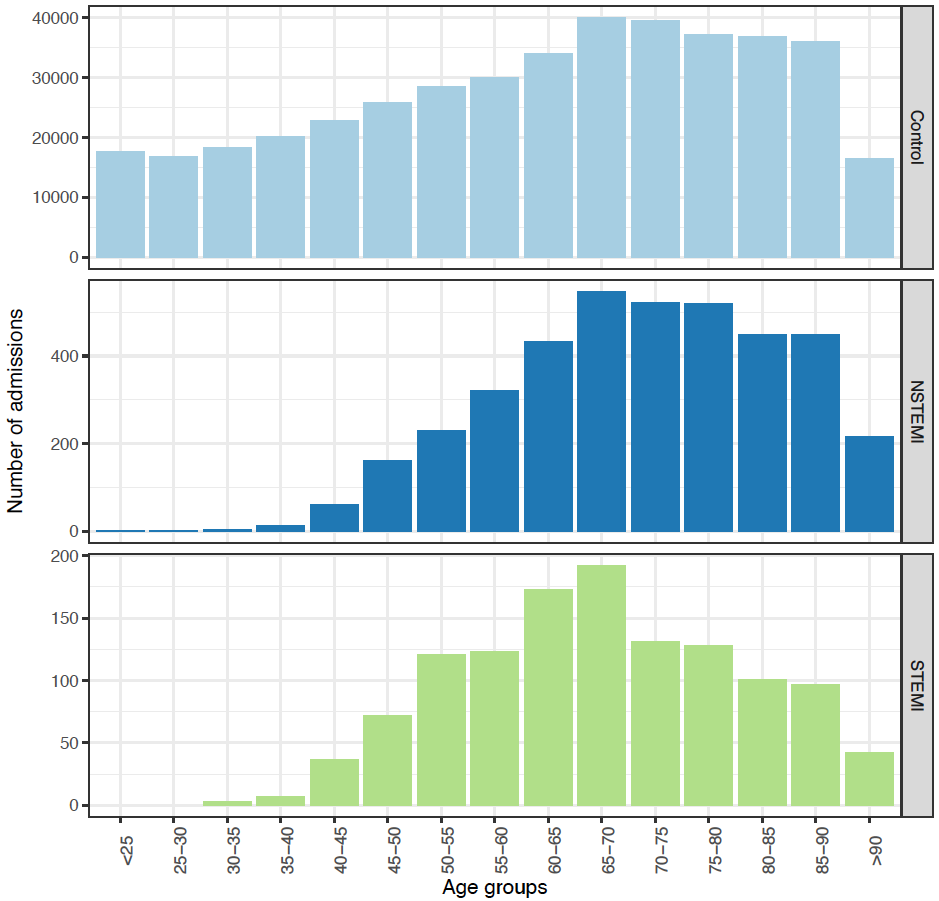


Note the different y-scale in different panels.

**Supplementary Figure 2.** Distributions of ECG date (year and quarter bins), by control/NSTEMI/STEMI status.


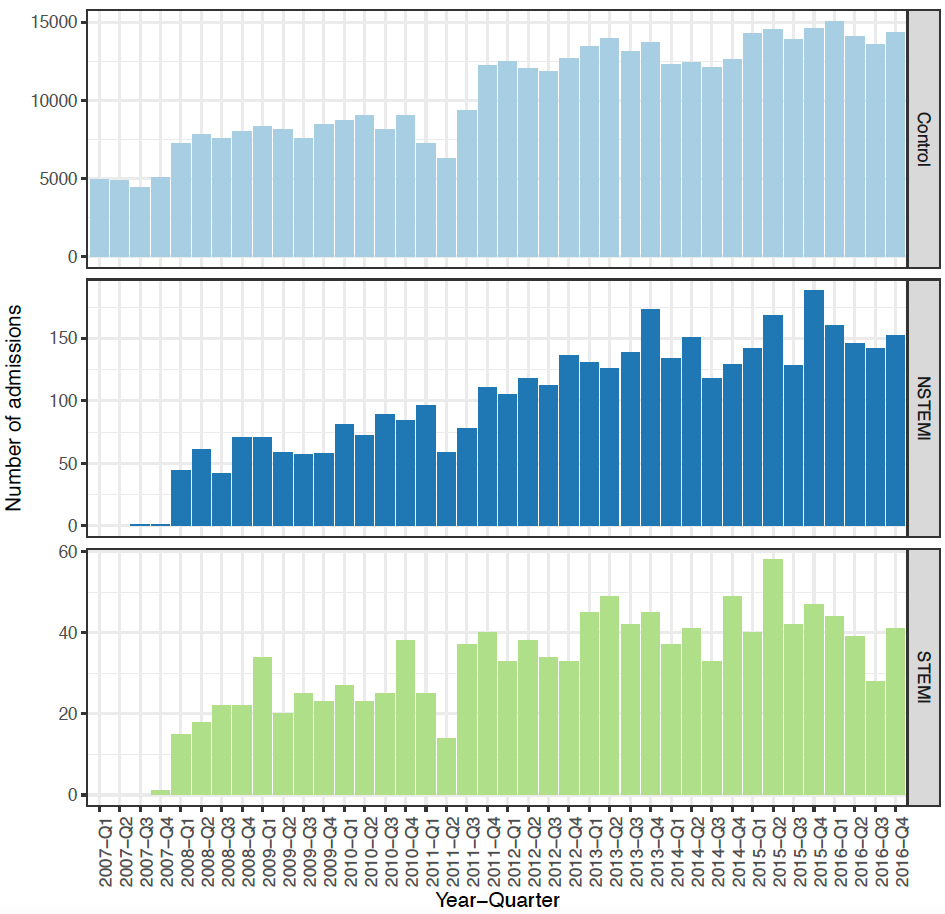


Note the different y-scale in different panels.

**Supplementary Figure 3.** Calibration of the final model.

Calibration plot for NSTEMI vs all and STEMI vs all for both of our test sets (random and temporal). Blue solid lines are LOESS (local regression) smooths of the estimated probabilities *versus* the class membership accompanied by a logistic calibration curve in green (dotted) resulting from regressing the estimated logits on the class membership. The light-blue shaded areas are 95% bootstrap confidence intervals from a bootstrap with 2,000 replicates. The black solid lines indicate ideal calibration. Intercept denotes the intercept from the logistic models and should be as close to zero as possible. Slope denotes the slope of the logistic regression fit and should be as close to 1 as possible. A slope > 1 indicates underfitting and a slope < 1 indicates overfitting with the degree of under-/overfitting directly proportional to the absolute size of the slope. Eavg, E90 and Emax correspond to the average absolute error, the 90th percentile of the absolute error and the maximum absolute error between the predicted probabilities and the LOESS fit. We tried to improve upon the original calibration by temperature scaling,^11^ vector calibration,^11^ and Dirichlet calibration.^12^ None of those methods succeeded in improving the model calibration.

**Supplementary Figure 4.** Predicted probabilities of STEMI for ECGs labeled as STEMI (top three panels), or for NSTEMI for ECGs labeled as NSTEMI (bottom three panels).


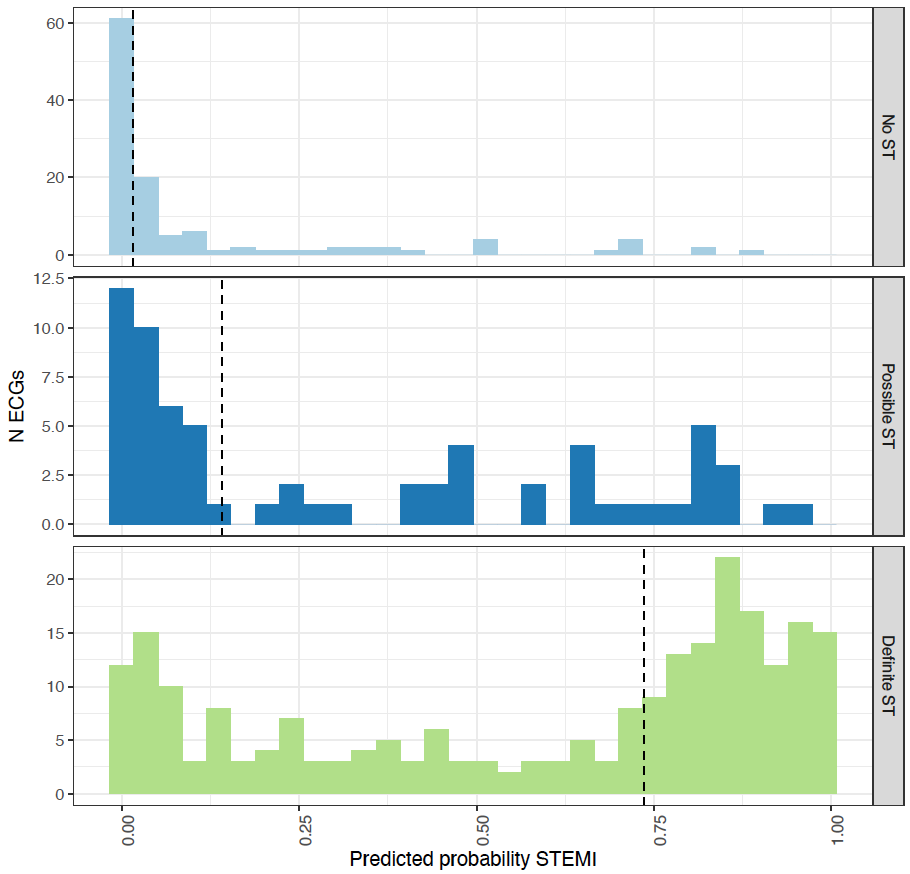


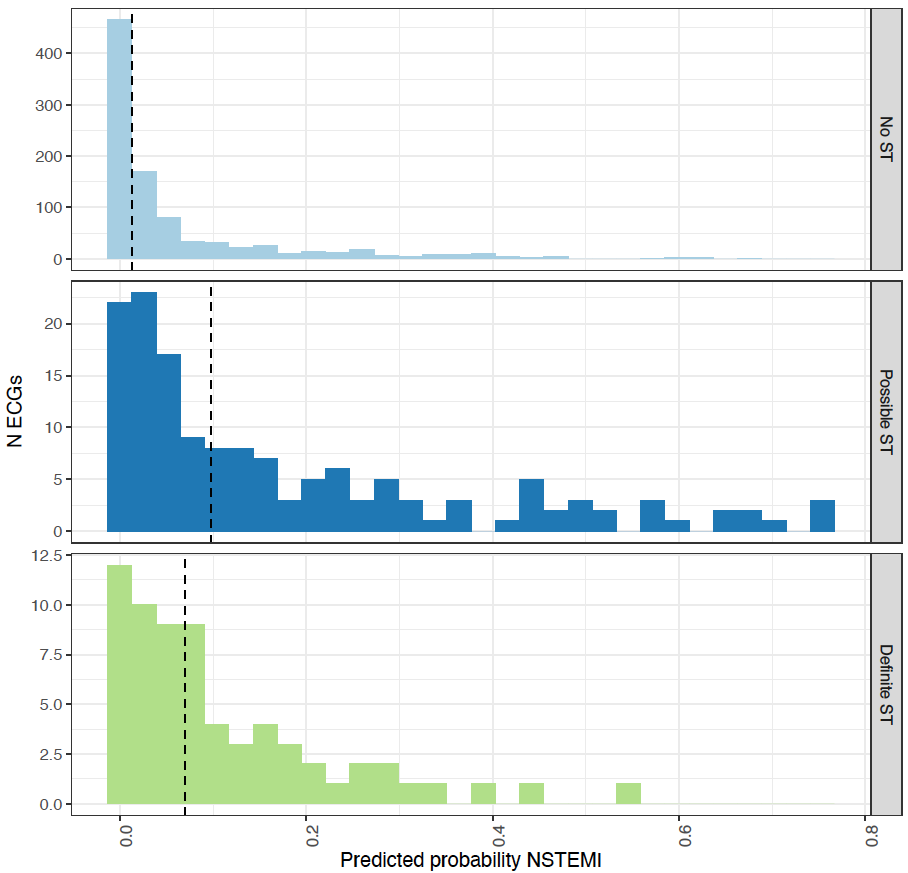


A manual evaluation of the presence of a ST-elevation was performed for the ECGs in the test set as described in the methods (no/possible/definite ST-elevation). The results are stratified by these groups and the median predicted probability of each group is represented by a vertical dashed line.

**Supplementary Figure 5.** C-statistics of STEMI *vs* rest (green) as well as NSTEMI *vs* rest (blue) in the temporal and random test sets, in subsets of patients.

##

The x-axis classes are given in the order: test set records without any filter applied based on ST-elevation label noise; ST-elevation filter corresponding to results in main table (used in all following test set subsets); stricter ST-elevation where “possible STEMI” is removed from all classes; age tertiles; sex; was the ECG collected at the same day as the admission or not ; did the patient visit the emergency department at Karolinska Hospital (main source of data) or another emergency department in the Stockholm region, patients attending the CCU only, ECGs recorded using the most common machine type (MAC55) or not, ECGs recorded using the most common software (v237) or not. Median (min-max) is presented from the model initiated with 10 random seeds.

**Supplementary Figure 6.** Loss and learning rate over epochs.


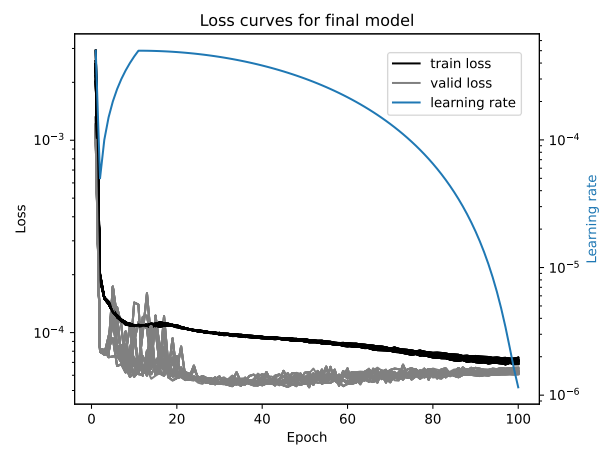
The validation loss in grey and training loss in black for all models, i.e. all ensemble members. Note that the validation loss is lower here due to the use of weight decay regularization and dropout during training but not during validation which is standard practice. We select the model with the lowest validation loss. The learning rate is a cosine annealing learning rate scheduler with linear warm up for the first 10 epochs. The y-axis in log mode distorts the cosine annealing function but is better suited to see the actual learning rate value at any given epoch.

# Supplementary References

1. Szummer K, Wallentin L, Lindhagen L, Alfredsson J, Erlinge D, Held C, James S, Kellerth T, Lindahl B, Ravn-Fischer A, Rydberg E, Yndigegn T and Jernberg T. Improved outcomes in patients with ST-elevation myocardial infarction during the last 20 years are related to implementation of evidence-based treatments: experiences from the SWEDEHEART registry 1995-2014. *Eur Heart J*. 2017;38:3056-3065.

2. Szummer K, Wallentin L, Lindhagen L, Alfredsson J, Erlinge D, Held C, James S, Kellerth T, Lindahl B, Ravn-Fischer A, Rydberg E, Yndigegn T and Jernberg T. Relations between implementation of new treatments and improved outcomes in patients with non-ST-elevation myocardial infarction during the last 20 years: experiences from SWEDEHEART registry 1995 to 2014. *Eur Heart J*. 2018;39:3766-3776.

3. Af Ugglas B, Djarv T, Ljungman PLS and Holzmann MJ. Association Between Hospital Bed Occupancy and Outcomes in Emergency Care: A Cohort Study in Stockholm Region, Sweden, 2012 to 2016. *Ann Emerg Med*. 2020;76:179-190.

4. SWEDEHEART variable list, <https://www.ucr.uu.se/swedeheart/dokument-sh/variabellista>.

5. Jernberg T, Attebring MF, Hambraeus K, Ivert T, James S, Jeppsson A, Lagerqvist B, Lindahl B, Stenestrand U and Wallentin L. The Swedish Web-system for enhancement and development of evidence-based care in heart disease evaluated according to recommended therapies (SWEDEHEART). *Heart*. 2010;96:1617-21.

6. Ribeiro AH, Ribeiro MH, Paixao GMM, Oliveira DM, Gomes PR, Canazart JA, Ferreira MPS, Andersson CR, Macfarlane PW, Meira W, Jr., Schon TB and Ribeiro ALP. Automatic diagnosis of the 12-lead ECG using a deep neural network. *Nat Commun*. 2020;11:1760.

7. Hu J, Shen L and Sun G. Squeeze-and-Excitation Networks. *2018 IEEE/CVF Conference on Computer Vision and Pattern Recognition*. 2018:7132-7141.

8. Bello I, Fedus W, Du X, Cubuk ED, Srinivas A, Lin T-Y, Shlens J and Zoph B. Revisiting resnets: Improved training and scaling strategies. *arXiv preprint arXiv:210307579*. 2021.

9. He T, Zhang Z, Zhang H, Zhang Z, Xie J and Li M. Bag of tricks for image classification with convolutional neural networks. *Proceedings of the IEEE/CVF Conference on Computer Vision and Pattern Recognition*. 2019:558-567.

10. Liu L, Jiang H, He P, Chen W, Liu X, Gao J and Han J. *On the Variance of the Adaptive Learning Rate and Beyond*; 2019.

11. Guo C, Pleiss G, Sun Y and Weinberger KQ. On calibration of modern neural networks. *International Conference on Machine Learning*. 2017:1321-1330.

12. Kull M, Perello-Nieto M, Kängsepp M, Song H and Flach P. Beyond temperature scaling: Obtaining well-calibrated multiclass probabilities with Dirichlet calibration. *Advances in Neural Information Processing Systems*. 2019:12295–12305.

13. Wagner P, Strodthoff N, Bousseljot RD, Kreiseler D, Lunze FI, Samek W and Schaeffter T. PTB-XL, a large publicly available electrocardiography dataset. *Sci Data*. 2020;7:154.

14. Goldberger AL, Amaral LA, Glass L, Hausdorff JM, Ivanov PC, Mark RG, Mietus JE, Moody GB, Peng CK and Stanley HE. PhysioBank, PhysioToolkit, and PhysioNet: components of a new research resource for complex physiologic signals. *Circulation*. 2000;101:E215-20.

15. Hothorn T, Hornik K, van de Wiel MA and Zeileis A. Implementing a Class of Permutation Tests: The coin Package. *Journal of Statistical Software*. 2008;28:1 - 23.
